# Fatal Cerebral Air Embolism Following Central Venous Catheter Mishandling in a Stroke Patient: A Case Report

**DOI:** 10.1155/crcc/6367510

**Published:** 2026-06-20

**Authors:** Lucas Ivan Sebastian Rundblad, Troels Gil Lukassen, Anna Garcia-Alix Grynnerup, Sura Azhar Abdulmunem, Helle Klingenberg Iversen, Anders Sode West

**Affiliations:** ^1^ Clinical Stroke Research Unit, Department of Neurology, University Hospital Rigshospitalet, Glostrup, Denmark, rigshospitalet.dk; ^2^ Department of Anesthesiology, University Hospital Rigshospitalet, Copenhagen, Denmark, rigshospitalet.dk; ^3^ Department of Radiology, Zealand University Hospital, Roskilde, Denmark, regionsjaelland.dk; ^4^ Department of Clinical Medicine, Faculty of Health and Medical Sciences, University of Copenhagen, Copenhagen, Denmark, ku.dk

**Keywords:** air embolism, central venous catheter, hemodialysis, stroke

## Abstract

**Background:**

Air embolism is a rare but potentially fatal complication associated with central venous catheter (CVC) use even several days after placement. Although often asymptomatic, large or rapidly introduced volumes of air can result in severe neurologic and cardiovascular compromise.

**Case Presentation:**

We report a case of fatal cerebral air embolism in a male stroke patient in his early 60s with a history of diabetes, nephropathy, and peritoneal dialysis. Following ischemic stroke, a CVC was inserted for hemodialysis. Seventeen days after stroke onset, the patient was found unconscious, with seizures and signs of acute neurologic deterioration. The hemodialysis catheter was discovered unplugged and unclamped, suggesting accidental or intentional self‐manipulation. Imaging confirmed a massive air embolism in both arterial and venous cerebral circulations. Despite immediate intervention, the patient remained unconscious and died 5 days later. Autopsy confirmed the diagnosis and excluded cardiac or pulmonary shunts.

**Conclusion:**

This case highlights the fatal potential of air embolism following CVC mishandling. Prevention requires meticulous catheter management, staff training, and informing patients about risks.

## 1. Introduction

Air embolism is a rare but potentially fatal event. Its development requires the introduction of air into the vascular system, which can occur during trauma, surgical procedures, or via intravascular catheters. In addition, a pressure gradient favoring the entry of air into the circulation—such as that caused by hypovolemia, deep inspiration, or surgical interventions performed above the level of the heart—is typically necessary for air embolism to occur [1].

It is presumed that most air embolism episodes are asymptomatic, whereas clinical symptoms are related to the speed and amount of air introduced [2]. Symptomatic air embolism carries a high mortality and morbidity, but is preventable [3, 4].

The occurrence of venous air embolism related to daily handling of central venous catheters (CVCs) has been reported to be as high as 17.2%, with central venous catheterization implicated in approximately one‐third of all cases [5]. However, the true incidence is likely underestimated due to the frequent absence of symptoms [2]. Symptomatic air embolism can be fatal, with reported mortality rates up to 21% and considerable associated morbidity [3, 6]. When an autopsy is performed, air embolism can be difficult to verify due to prior absorption of air.

Arterial air embolism occurs when air enters the arterial system directly or via paradoxical embolization through a cardiac defect such as a patent foramen ovale (PFO) or a pulmonary arteriovenous malformation [7]. Furthermore, a high rate of venous air may exceed the pulmonary filtration capacity and proceed as arterial air emboli [1, 8]. Once in the arterial circulation, air emboli obstruct blood flow leading to end‐organ ischemia. If air reaches the cerebral circulation, there is a high risk of morbidity and mortality [7, 9]. Air introduced into the venous system can also ascend retrogradely through the jugular veins due to its buoyancy, lodge in the cerebral sinuses and cortical veins, and cause cerebral venous infarction [1, 2, 4].

The clinical presentation of cerebral air embolism, of both venous and arterial origin, may include seizures, focal neurological deficits, and altered mental status [4, 9]. Large volumes of air induced into the venous system can create an “air lock” in the heart′s right ventricular outflow tract, obstructing pulmonary circulation and resulting in acute right heart failure. This may manifest clinically as dyspnea, pulmonary hypertension, arrhythmias, and hemodynamic collapse [1, 2, 7].

We report a fatal case of air embolism, secondary to mishandling of a CVC for hemodialysis.

## 2. Patient Information

**Figure 1:**
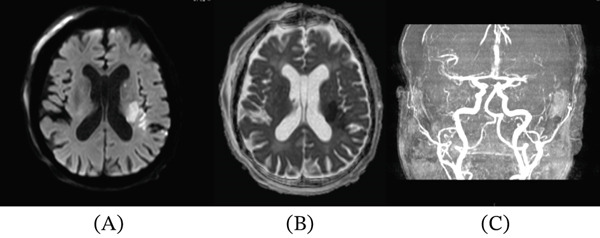
Diffusion‐weighted imaging (DWI) reveals areas of diffusion restriction in the vascular territory of the left middle cerebral artery (A) with corresponding low apparent diffusion coefficient (ADC) values (B), suggesting the acute ischemia caused by the occlusion of the left medial cerebral artery, distal M1 segment/proximal M2 branches with corresponding lack of flow voids on arterial time of flight (TOF) sequence (C).

A man in his early 60s with a medical history of arterial hypertension, chronic alcohol misuse, diabetes mellitus, and diabetic nephropathy, undergoing peritoneal dialysis at the time of admission. He was admitted to the stroke unit following the sudden onset of aphasia and right‐sided hemiparesis. A magnetic resonance imaging (MRI) of the brain confirmed ischemic stroke (Figure 1). Due to the severity of his neurological deficits, the patient was unable to continue peritoneal dialysis, and a 13 Fr (4.3 mm) CVC was inserted into the right internal jugular vein to facilitate hemodialysis.

Seventeen days after stroke onset, and 13 days following CVC placement, the patient was found unconscious in his wheelchair in the dining area of the rehabilitation ward, exhibiting leftward gaze deviation and generalized tonic muscle contractions. Minutes earlier, he had been outside smoking with acquaintances. The hemodialysis catheter was discovered without its plugs and clamps, with the dressing removed and the protective patch peeled off. These items were later found on the floor of the dining area. Initially, no backflow of blood was observed from the CVC, but upon repositioning the patient to a horizontal position in bed, backflow of blood was noted. The patient subsequently developed generalized convulsions, and intravenous treatment with 12.5 mg diazepam and 4000 mg levetiracetam was administered.

As the CVC had been verified to be properly secured earlier the same day, initial concerns were raised regarding the possibility of a criminal act. However, video surveillance showed no evidence of foul play. Furthermore, the patient′s medical records included a statement indicating that he was tired of living and did not wish to continue dialysis. Consequently, it was concluded that the patient had likely tampered with the CVC himself shortly after returning to the ward, possibly unaware of the consequences of his action.

## 3. Clinical Findings and Follow‐Up

**Figure 2:**
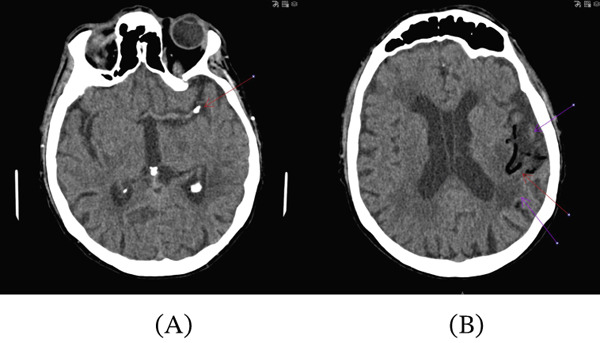
Computed tomography (CT) of the brain in soft tissue window (A and B), performed within 1 h after the event, showing the dense artery sign (red arrow in A) corresponding to the earlier known occluded segment of the middle cerebral artery with surrounding hypodensities (purple arrows in B) representing demarcation of ischemic changes. It also reveals the new finding of arterial air embolism along M2 and M3 branches (red arrow in B) of the left middle cerebral artery.

**Figure 3:**
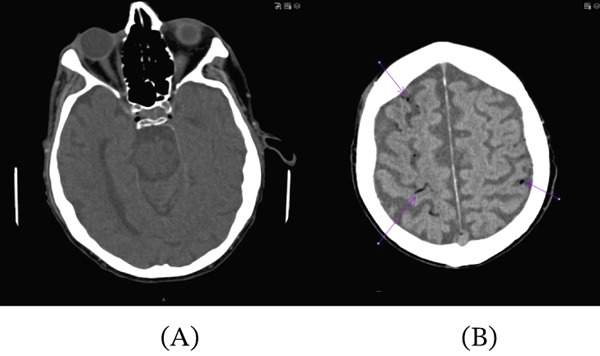
Computed tomography (CT) of the brain, adjusted towards lung window (A and B). Reveals air bobbles on both sides of the cavernous sinus (A) and shows the small air emboli (purple arrows in B) involving the leptomeningeal venules and arterioles along the subarachnoid spaces on both cerebral hemispheres.

A subsequent CT scan of the brain, performed within 1 h, revealed the presence of air within both sides of the cavernous sinuses, as well as along the occluded segment of the left middle cerebral artery and bilaterally along the cortical vessels, consistent with the coexistence of both arterial and venous air embolism (Figures 2 and 3). The patient remained unconscious but continued to breathe spontaneously. Life‐sustaining treatment was withdrawn, and the patient passed away 5 days following the event. No MRI was performed after the event.

Autopsy findings included ischemic changes in the myocardium and bilateral cerebral lesions, with evidence of recent ischemia in the right hemisphere and slightly older ischemic changes in the left. There were no indications of a PFO in the heart, and the lungs were free from arteriovenous malformations. The hemodialysis catheter was subsequently evaluated by two experienced anesthesiologists, who found it to be well‐functioning and without visible defects. Their assessment concluded that the catheter′s opening must have been intentional. The timeline of events is shown in Table 1.

**Table 1:** Timeline.

Day	Event
0	Patient admitted to stroke unit with acute aphasia and right hemiparesis. MRI showed ischemic stroke.
4	Central venous catheter inserted in the right internal jugular vein.
7	First hemodialysis performed.
15	Patient transferred to stroke rehabilitation ward.
17	Patient found unconscious with seizures in wheelchair. Central venous catheter showed signs of mishandling. Imaging revealed cerebral air embolism.
22	Patient died. Autopsy confirmed bilateral brain ischemia and no cardiac or pulmonary shunts.

## 4. Discussion

Cerebral air embolism is a rare but potentially devastating complication associated with manipulation of CVCs [10]. Although some recent reports emphasize the prerequisite presence of a venous‐to‐arterial shunt, most commonly a PFO, to allow paradoxical embolization [11], our patient demonstrated no evidence of right‐to‐left shunting on autopsy. The occurrence of cerebral air embolism in the absence of intracardiac shunting has been previously reported. Song et al. [12] described a 57‐year‐old patient who developed cerebral air embolism following removal of a CVC despite the absence of structural cardiac abnormalities, suggesting that alternative mechanisms can permit air entry into the cerebral circulation.

These clinical observations are consistent with established mechanisms by which air may be introduced into the cerebral vasculature during CVC manipulation by healthcare professionals. However, as highlighted by the present case, patients may also be at risk during hospitalization outside of procedural settings, when vigilance regarding CVC safety may be reduced. Ozair et al. [13] similarly reported that lapses in catheter care and handling during routine inpatient management can contribute to the development of air embolism, underscoring the importance of continuous awareness and adherence to preventive measures throughout the entire duration of catheter use.

CT is the first‐line imaging modality in patients presenting with acute neurological deficits and when cerebral air embolism is suspected. It allows direct visualization of intracranial air and enables rapid exclusion of alternative causes of acute neurological deterioration. However, the diagnostic sensitivity of CT is highly time‐dependent due to rapid resorption of intracranial air. Air is optimally detected within the first 1–1.5 h after symptom onset and may completely disappear within approximately 16 h [14].

When CT is delayed or negative, MRI has been shown to be valuable in demonstrating secondary parenchymal injury rather than direct visualization of intracranial air. MRI findings in cerebral air embolism are nonspecific and may resemble infarcts of various arterial or venous etiologies. As described by Timpone and Callen [15], typical MRI features include a mixed pattern of cytotoxic and vasogenic edema, with areas of restricted diffusion on diffusion weighted imaging (DWI)/apparent diffusion coefficient (ADC) accompanied by fluid‐attenuated inversion recovery (FLAIR) hyperintensity. These changes may demonstrate a border‐zone distribution and can be associated with leptomeningeal enhancement, reflecting blood–brain barrier disruption.

In our patients′ case, the CT scan was obtained within 1 h after symptom onset and showed air emboli in cavernous sinuses and cerebral arteries, suggesting both retrograde venous and arterial cerebral air embolism (Figures 2B and 3), as well as a demarcated infarct related to a prior arterial occlusive event (Figure 2B). MRI was not feasible. Postmortem examination confirmed older ischemic changes in the left cerebral hemisphere and more recent ischemic changes in the right hemisphere, retrospectively supporting the CT findings and the expected evolution of parenchymal injury leading to a fatal outcome.

Retrograde venous air embolism is believed to be facilitated by the gravity of air, allowing air bubbles to ascend against venous blood flow, particularly in upright patients [16]. This mechanism explains the presence of air within the cerebral venous system, including the cavernous sinuses, as observed in this case. In contrast, arterial cerebral air embolism could be explained by a paradoxical air embolism, that is, venous air entering the arterial circulation through a right‐to‐left shunt, most commonly a PFO or an arteriovenous malformation in the lungs [4]. However, the autopsy did not identify any intracardiac shunt or arteriovenous malformation, making this mechanism unlikely in the present case. Thus, transpulmonary passage of air through an overwhelmed pulmonary filter is the most plausible explanation for the concomitant arterial cerebral air embolism observed in our patient. It is well‐described that large volumes and/or rapid entrainment of air may exceed the filtering capacity of the pulmonary circulation, allowing air to pass into the arterial system even in the absence of a structural shunt [17]. The mishandling of the CVC was not directly observed, but most likely, the patient was sitting up in a wheelchair when the catheter was opened. The duration for which the catheter remained open before the patient was discovered is unknown. It is presumed that the combination of negative intrathoracic pressure due to the patient′s upright posture and the large catheter size (13 Fr) facilitated the massive entry of air.

The rate of air entrainment depends on the pressure difference across the catheter and its internal diameter. This rate is clinically significant, as the pulmonary circulation and alveoli serve as reservoirs for removing intravascular gas. If the rate of entry is slow, the heart and lungs may be able to cope with large volumes of air over a prolonged period, as shown in animal studies [18]. Experimental studies have shown that a pressure decrease of 5 cm H_2_O across a 14‐gauge needle (internal diameter of 1.8 mm) can transmit approximately 100 mL of air per second [19]. The fatal dose in humans is unknown, but based on animal studies and case‐reports, the estimates vary between 300 and 500 mL at 100 mL of air/s. Thus, an open CVC with an internal diameter of 4.3 mm in a patient with negative thoracic pressure due to spontaneous breathing in the upright position, as in the presented case, would easily cause a lethal accumulation of air if not addressed immediately.

Management of air embolism involves prompt identification of the source of air entry and immediate prevention of further air entry, in addition to supportive care aimed at increasing intravascular volume and venous pressure. The initial intervention for both arterial and venous air emboli is the administration of high‐flow 100% oxygen, which establishes a diffusion gradient that enhances pulmonary nitrogen washout and reduces the volume of the embolism [1, 2]. Symptomatic treatment, including the administration of anticonvulsants, may also be required [1, 7].

In cases of large venous air emboli lodging within the right ventricle of the heart, the Trendelenburg position combined with left lateral decubitus positioning (“Durant′s maneuver”) has been suggested to shift the air to the right ventricular apex due to buoyancy. However, there is no clear evidence to support this. Chest compression could cause frothing of the air and absorption via the pulmonal filtration capacity [1, 2]. Intracardiac aspiration of air using different types of catheters has also been attempted in cases with large intracardial air emboli; however, results are mixed, and the procedure is used infrequently [1, 2, 7]. If a catheter is already in place, aspiration of air from the right atrium should be considered as a first‐line intervention [2].

When available and logistically feasible, hyperbaric oxygen therapy (HBOT) is considered the primary treatment of cerebral air embolism. HBOT enhances the dissolution of intravascular gas, promotes nitrogen washout, and reduces embolus volume. Additionally, it induces hyperoxia, increasing oxygen delivery to ischemic brain tissue [1, 7]. Early initiation of HBOT yields the best outcomes; however, clinical benefit has been reported even when treatment is commenced more than 48 h after symptom onset. The major limitation of HBOT is the need for patient transport to specialized facilities, which may pose considerable risks for hemodynamically unstable patients [1, 2].

In our case, treatment included elimination of the air entry point by closing the catheter and anticonvulsive therapy. Due to the patient′s severe pre‐existing conditions, transfer to a hyperbaric oxygen facility was deemed futile.

In conclusion, this case underscores air embolism as a rare but potentially fatal complication to CVC mishandling. The combination of a wide diameter of the catheter and the patient′s upright position likely led to a massive influx of air resulting in venous air emboli that overwhelmed the normal pulmonal filtration and proceeded as arterial air emboli, leading to cerebral infarction and death. This case highlights the importance of cautious monitoring of CVCs and the necessity of educating both patients and healthcare providers about the risks of air embolism.

## Author Contributions

L.I.S.R. collected the data, elaborated the manuscript, and critically edited the manuscript. T.G.L. collected the data, drafted the original manuscript, and critically edited the manuscript. A.G‐A.G. elaborated part of the manuscript, provided anesthesiologic expertise, and critically edited the manuscript. S.A.A. elaborated part of the manuscript, collected the data, provided neuroradiology expertise, and critically edited the manuscript. H.K.I. provided stroke expertise and critically edited the manuscript. A.S.W. elaborated part of the manuscript, provided stroke expertise, and critically edited the manuscript. L.I.S.R and T.G.L have contributed equally to this work and share first authorship.

## Funding

No funding was received for this manuscript.

## Consent

The patient was unable to provide informed consent and had no known living relatives. Permission to publish the case was obtained through the legal department of University Hospital Rigshospitalet.

## Conflicts of Interest

The authors declare no conflicts of interest.

## Data Availability

Data sharing is not applicable to this article as no datasets were generated or analyzed during the current study.
